# Triboelectric Nanogenerators: State of the Art

**DOI:** 10.3390/s24134298

**Published:** 2024-07-02

**Authors:** Zhan Shi, Yanhu Zhang, Jiawei Gu, Bao Liu, Hao Fu, Hongyu Liang, Jinghu Ji

**Affiliations:** 1 School of Mechanical Engineering, Jiangsu University, No. 301 Xuefu Road, Zhenjiang 212013, China; shizhan12101999@163.com (Z.S.); 2222203096@stmail.ujs.edu.cn (J.G.); fh@ujs.edu.cn (H.F.); hyliang@ujs.edu.cn (H.L.); jijinghu@ujs.edu.cn (J.J.); 2 Institute of Advanced Manufacturing and Modern Equipment Technology, Jiangsu University, No. 301 Xuefu Road, Zhenjiang 212013, China; 3 Institute of Automotive Engineering, Jiangsu University, No. 301 Xuefu Road, Zhenjiang 212013, China; liubao921@163.com

**Keywords:** triboelectric nanogenerator, research status, application development, performance improvement

## Abstract

The triboelectric nanogenerator (TENG), as a novel energy harvesting technology, has garnered widespread attention. As a relatively young field in nanogenerator research, investigations into various aspects of the TENG are still ongoing. This review summarizes the development and dissemination of the fundamental principles of triboelectricity generation. It outlines the evolution of triboelectricity principles, ranging from the fabrication of the first TENG to the selection of triboelectric materials and the confirmation of the electron cloud overlapping model. Furthermore, recent advancements in TENG application scenarios are discussed from four perspectives, along with the research progress in performance optimization through three primary approaches, highlighting their respective strengths and limitations. Finally, the paper addresses the major challenges hindering the practical application and widespread adoption of TENGs, while also providing insights into future developments. With continued research on the TENG, it is expected that these challenges can be overcome, paving the way for its extensive utilization in various real-world scenarios.

## 1. Introduction

The development of human society and economic growth are intricately dependent on energy, which forms the cornerstone of a nation’s position in the world. However, the extraction of fossil fuels (e.g., oil, natural gas, and coal) is finite. Unrestricted extraction will deplete resources, lead to environmental problems, and jeopardize the Earth’s ecological balance. Hitherto, the quest for stable, green, sustainable, and efficiently utilized alternative energy sources is a pressing issue facing contemporary society. Despite research advancements in the gradual conversion of solar and wind energy into electricity, factors like environmental pollution and high costs have gradually constrained further development in this area.

Today, electronic products are gradually developing towards miniaturization and are widely distributed in various aspects of our lives, with a wide range of functions, such as health monitoring, environmental monitoring, infrastructure monitoring, security checks, and more. The components of electronic products have been required to become increasingly miniaturized and energy storage components, such as batteries, are also subject to higher demands, smaller while storing more energy, which adds complexity to the battery industry. The miniaturization of batteries requires greater investment in human resources, material resources, and effort for research. Moreover, batteries generate pollution in the environment after use, necessitating significant expenses to address battery pollution [1]. Therefore, developing new clean and efficient renewable energy sources, further miniaturized to fit microsensor networks, has become a matter of great concern across various sectors of society.

However, there exists a significant amount of wasted energy in our daily lives, tiny mechanical energy. In various movements such as vibration, rotation, and displacement, tiny mechanical energy is often wasted rather than effectively utilized. Collecting and converting it into usable energy is an ideal approach to energy harvesting. Electromagnetic and piezoelectric generators are well-known as excellent energy conversion devices; yet, they still face unavoidable challenges. Despite efficiently harnessing various ambient mechanical energy forms of electromagnetic generators, the limitations of large and heavy coils and magnets restrict their flexibility and portability [2,3,4,5,6,7]. Piezoelectric generators exhibit excellent sensitivity to mechanical stimuli and natural advantages in operating at high frequencies. However, they are constrained by the choice of piezoelectric materials and operating modes [8,9,10,11,12]. Triboelectric nanogenerators (TENGs) have also emerged as novel nanogenerators that harvest mechanical energy from the environment. In comparison, TENGs have a simple structure, are compact, lightweight, and portable, and offer a variety of choices for friction layer materials. Additionally, TENGs with multiple operating modes can easily adapt to different application scenarios and efficiently collect overlooked weak mechanical energy conversions from the environment and bodily movements into electrical output [13,14,15,16,17,18,19].

This review summarized the widely accepted working principles of triboelectric nanogenerators (TENGs) and their developmental research progress. We provided an overview of the current status of TENG development, including working modes (with their strengths and weaknesses) and material selection. Additionally, numerous researchers have been dedicated to realizing specific applications of TENGs in real-life scenarios and have made various attempts. We categorized and summarized these attempts based on different application scenarios. Addressing one challenge faced by TENGs, namely, weak output performance, especially in terms of current output, we compiled and discussed various research directions for performance optimization currently being pursued. We hope this review will offer valuable insights, fostering a deeper understanding and encouraging further investigations in this field, thereby contributing significantly to the ongoing development of triboelectric nanogenerators.

## 2. Overview of Triboelectric Nanogenerator

### 2.1. Electrical Principles

**Figure 1:**
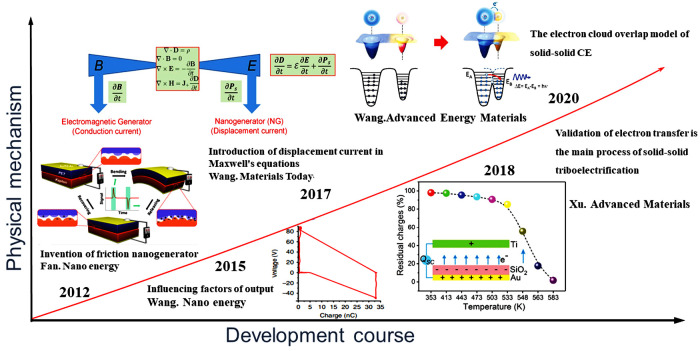
Evolutionary progress of the triboelectric charging mechanism in TENGs [20,21,22,23,24].

The phenomenon of triboelectric charging is not unfamiliar to humans, and its records date back to over 2600 years ago in ancient Greek civilization. Static electricity phenomena are also commonly observed in daily life. However, its physical mechanism and principles have long remained unclear. In 2012, a team led by Zhong Lin Wang invented the triboelectric nanogenerator (TENG) based on the triboelectric charging phenomenon and the principle of electrostatic induction (Figure 1) [20]. In 2015, Wang et al. established a theoretical model for the TENG, determining several important parameters: transferred charge *Q*, open-circuit voltage *V*, short-circuit current *I*, and gap distance *x* [21]. In 2017, their team introduced the Ps term brought by surface charges into the classical Maxwell displacement current equation, expanding the original equation to [22]:

(1) JD=ε0∂E∂t+∂Ps∂t

where *J_D_* is the displacement current, *D* is the displacement vector, *E* is the electric field, and *ε*_0_ is the vacuum permittivity. The displacement current consists of two terms. Unlike the Lorentz force driving electrons to flow in conductors, the second term expanded out (*∂P_s_*)/*∂t*, representing the displacement current driven by internal displacement within the generator, such as piezoelectric, thermoelectric, and triboelectric effects. With this, Professor Zhong Lin Wang expanded the first principle of the TENG based on Maxwell’s equations. In 2018, Xu et al. [23] demonstrated through real-time monitoring of charge output at high temperatures that electron transfer is the primary process in triboelectric charging and preliminarily proposed the electron cloud overlapping model. In 2020, the team led by Zhong Lin Wang summarized the contact electrification principles of objects in different states and formally proposed the microscopic process of contact electrification—the electron cloud overlapping model. The paper pointed out that the occurrence of contact electrification arises from external forces, and the electron clouds between two atoms overlap, resulting in a decrease in the potential barrier between them, ultimately leading to electron migration. Additionally, contact electrification occurs when the distance between two atoms is less than the bond length [24]. This model was supported by quantum mechanical calculations, which showed that the driving force for electron transfer is the delocalization of electron wave functions caused by contact and strain, which is the strong overlap state of the electron clouds referred to in this model under stress [25,26,27]. Within this range, electron transfer is possible, and the transition probability of electrons from one atom to another was calculated as a function of interatomic distance [28].

### 2.2. Operating Mode

**Figure 2:**
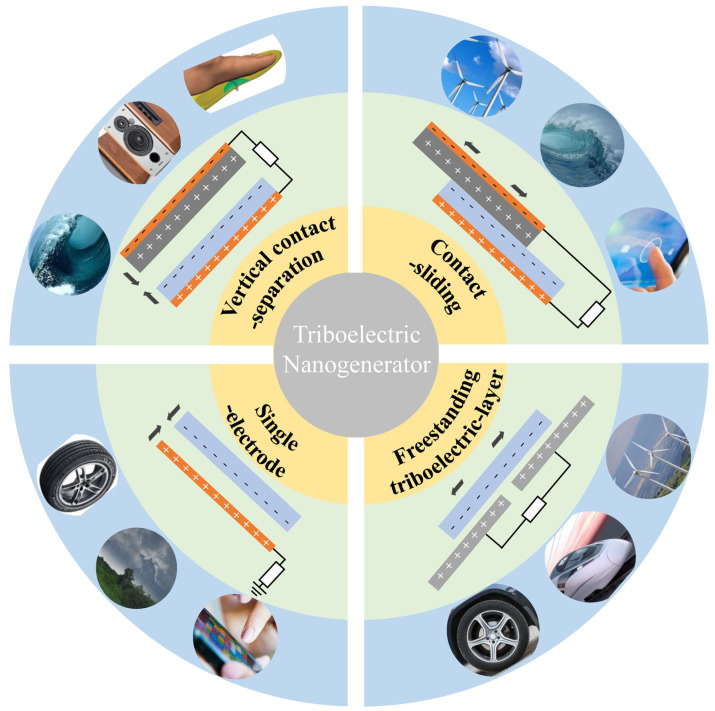
Four basic operating modes of TENG [29]: vertical contact-separation mode, horizontal sliding mode, single-electrode mode, and independent layer mode.

Based on the principle of frictional electricity generation, the electrical energy of a triboelectric nanogenerator (TENG) originates from the contact electrification of the friction layer, followed by electrostatic induction caused by the subsequent separation. Repeated contact and separation induce electron flow throughout the device, resulting in electrical output. Based on this contact-separation operation mode, TENG can derive four different fundamental working modes: vertical contact-separation mode, horizontal sliding mode, single-electrode mode, and independent-layer mode, as shown in Figure 2 [29].

(1) Vertical Contact-Separation Mode: The vertical contact-separation mode was the initial working mode used when the TENG was first invented, and it remains the most common mode of operation today. In this mode, two dielectric materials with different frictional charges (electron affinity) on their back surfaces are attached to metal electrodes. Under external force, they come into vertical contact, and charge transfer occurs, resulting in opposite charges on the two surfaces that reach equilibrium. When the two surfaces separate, the friction charges on both surfaces cannot reach equilibrium. By externally connecting the two metal electrodes, induced charges are generated on the back electrode due to electrostatic induction, causing electron flow in the circuit to balance the electrostatic field. Periodic contact separation generates pulsed alternating current, producing electrical output. TENGs operating in this mode have a relatively simple structure and strong shock resistance and can adapt to various frequencies of mechanical triggering, exhibiting high stability.

(2) Horizontal Sliding Mode: In the horizontal sliding mode, two materials with different frictional polarities slide horizontally against each other and electron transfer occurs during this process. Positive and negative charges will compensate for each other and reach equilibrium with the two fully aligned friction layers. The misaligned portion of charges cannot be compensated for, resulting in the formation of a potential difference. This promotes electron flow in the circuit to achieve equilibrium, thereby generating electrical output performance. Significant frictional loss will occur between the two friction layers in the horizontal sliding mode, which will greatly reduce the service life of the TENG. Therefore, when designing TENGs in the horizontal sliding mode, consideration must be given to the wear of the friction layers. However, the horizontal sliding mode provides ample frictional contact between the two friction layers, greatly improving the efficiency of charge separation and transfer. This has a significant impact on enhancing the performance of TENGs.

(3) Single-Electrode Mode: The single-electrode mode can be considered as a derivative mode of the vertical contact-separation mode. Compared to the vertical contact-separation mode, the single-electrode mode requires circuit connections between the two friction layers and their back electrodes, the single-electrode mode simplifies the setup and reduces packaging costs. In the single-electrode mode, only one electrode needs to be grounded, while the contact with the other friction layer allows surface friction charges to be obtained. During the cycle of separation and subsequent contact, electrons are alternately attracted from the ground and flow, generating alternating current for electrical output. This type of mode has a simpler structure and is easier to apply in scenarios where it is inconvenient to connect circuits between two electrode layers. However, it is important to note that this mode often results in lower output performance. Generally, the single-electrode mode is more suitable for various sensing and monitoring scenarios that only require weak currents for response, rather than direct applications in power generation and supply where higher output performance is required.

(4) Independent-Layer Mode: The independent-layer mode is derived from the horizontal sliding mode and involves a moving dielectric layer and two (or more) horizontal arrays of metal electrodes. The geometric shape of the moving friction layer needs to be consistent with the stationary metal electrodes in the horizontal arrays, with gaps left between the metal electrodes in the arrays. Generally, this mode can be pre-charged through the triboelectric effect before operation to ensure the generation of surface friction charges. Then, a certain air gap is left between the friction layer and the metal electrodes to allow for the generation of induced charges even when separated by a distance. As the active friction layer moves horizontally, the surface friction charges induce opposite charges on the metal electrodes. When the two layers are fully aligned, positive and negative charges on the surfaces reach equilibrium. However, as the active friction layer continues to slide and undergo electrostatic induction with the next metal electrode, the charge balance between the previous and next metal electrodes is disrupted. This creates a potential difference between the two metal electrodes, causing electrons to flow within the circuit between the two back electrodes to balance the local potential distribution. It is evident that due to the absence of direct contact, surface friction loss is minimal in the independent-layer mode, resulting in an extremely long lifespan. Therefore, the independent-layer mode combines high performance with longevity. The only drawback is that the design and circuit distribution of this mode are more complex, and its application scenarios are relatively limited compared to the vertical contact-separation mode.

### 2.3. Electrical Polarity of the Friction Layer Material

**Figure 3:**
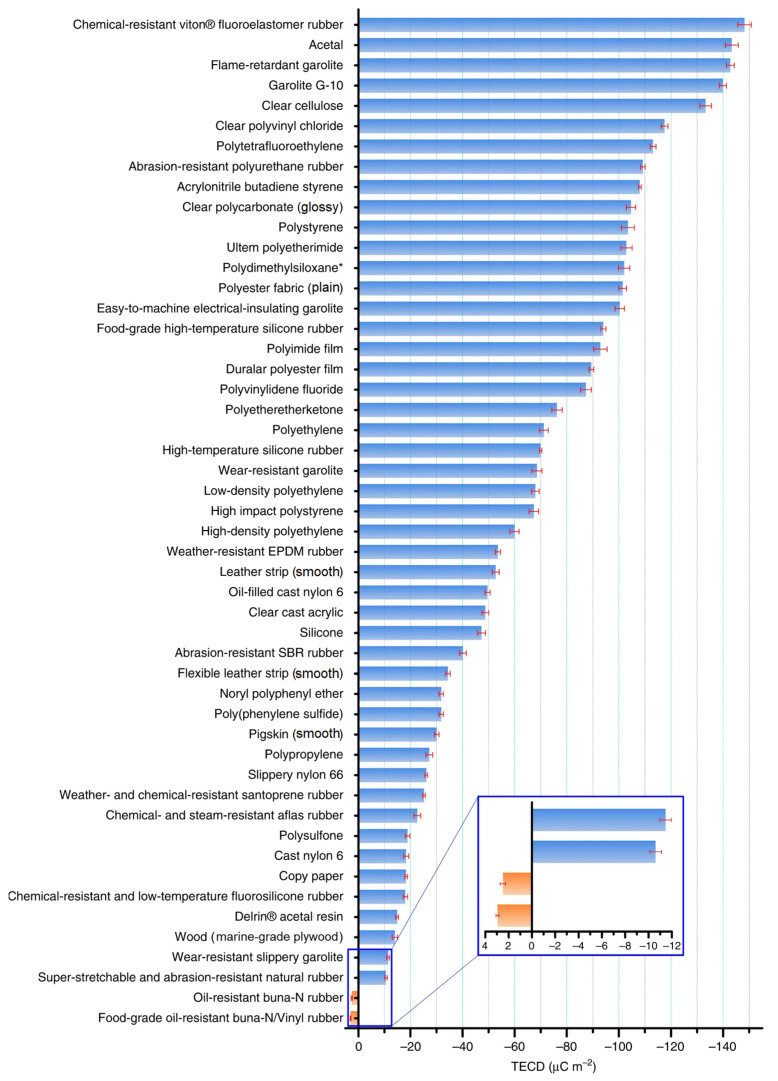
Triboelectric charge densities of series triboelectric materials [32]. The material marked with an asterisk “*” means it has strong adhesion with mercury, a small drop of mercury is observed when it is separated with mercury.

Due to the existence of multiple operating modes, TENG has a wide range of application scenarios, but the improvement of its performance is equally important. The contact performance of the two friction layers is the main factor affecting the performance of the TENG. Although theoretically, all materials can serve as friction layer materials for TENGs, the greater the difference in friction polarity between the two selected materials, the easier the electron transfer upon contact, resulting in a higher transfer charge, thereby obtaining a TENG with better performance. For the friction polarity of different types of materials, many researchers have measured surface charges and potentials during their triboelectric processes and, based on the results obtained, established a triboelectric series to guide the selection of friction layer materials during the preparation of the TENG [30,31]. However, the triboelectric performance is not only limited by the types of materials themselves but also affected by various environmental parameters, different contact mechanical parameters, and other factors, leading to deviations in experimental results among researchers. To more reasonably quantify the triboelectric performance of different materials, Zou et al. developed a method for measuring triboelectric performance using liquid metal-mercury, which systematically arranged the triboelectric series of various materials, as shown in Figure 3, providing a reference for the preparation of a TENG [32].

## 3. Current Status of Applied Research

**Figure 4:**
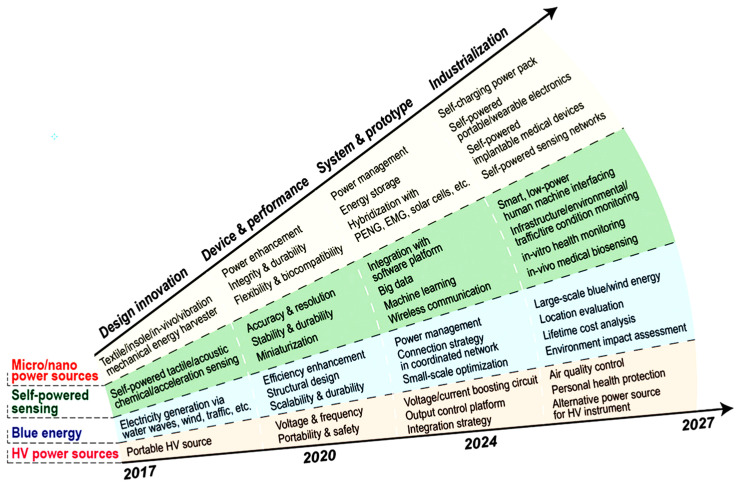
Development directions of TENGs in various applied scenarios [33].

Based on the working principles of a TENG and its four basic operating modes, various clever designs can be employed to construct the required generators and apply them to practical scenarios. Today, with the continuous exploration by scientists, the applications of TENGs have extended to many aspects of human daily life, production, activities, health, and more. Its application directions mainly fall into four categories: self-powered sensing, blue energy, micro/nano energy, and high-voltage power sources (Figure 4) [33].

### 3.1. Self-Powered Sensing

Due to its characteristic of converting mechanical signals into electrical signals, a TENG can autonomously generate electrical energy output based on specific frequencies and magnitudes of mechanical stimulation. Moreover, it only requires simple control circuits to achieve signal transmission. This natural advantage in assembly simplicity and the enormous potential for signal conduction make it highly applicable in the field of self-powered sensors.

**Figure 5:**
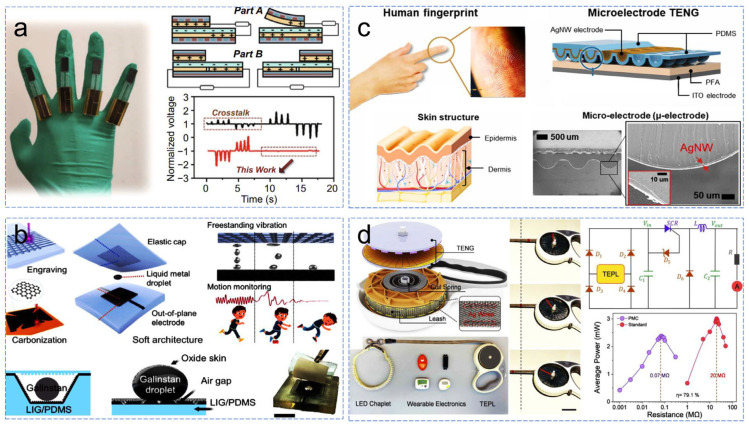
Self-powered sensing applications of TENG in various fields. (**a**) Real-time interactive self-powered TENG gesture signal sensor for operating a robotic hand, (**b**) self-powered TENG vibration signal sensor for monitoring tiny vibrations of liquid metal droplets, (**c**) fingerprint-inspired self-powered TENG pressure signal sensor for pressure monitoring, (**d**) self-powered TENG motion signal sensor for monitoring pet activity and health [34,35,36,37].

The triboelectric nanogenerator (TENG), with its various working modes and ability to self-power, can be designed as a variety of self-powered sensors (Figure 5) [34,35,36,37]. Typically, TENG gesture sensors convert different magnitudes of mechanical stimulation from finger movements or contact into corresponding electrical signals. However, this method inevitably results in signal instability due to the influence of various external factors [38,39,40]. To address this issue and design a sensor device that can interact in real-time with hand gestures and manipulate its operation while improving signal stability, Qin et al. [34] proposed a magnetic-array-assisted sliding triboelectric sensor (Ma-s-TS). This TENG operates primarily in sliding friction mode. The authors designed a magnetic-array-assisted sliding structure to constrain the sliding distance of the friction layer. This structure allows slider A to continuously switch between a repulsive and attractive state with the bottom magnet during sliding, thereby operating in both contact-separation and sliding friction modes. Slider B, with a different magnetic pole arrangement, remains in a consistently attractive state with the bottom magnet, operating independently in the sliding friction mode. These two sliders, or friction layers, output different types of electrical signals during operation: part A generates periodic narrow pulse signals due to alternating attraction/repulsion, while part B outputs wide pulse signals in the independent layer friction mode. These two signals are coupled to form positive/negative pulse signals corresponding to finger bending/straightening states (Figure 5). This coupling of finger bending into sliding friction mode and the resulting signal output allows for finer and more stable differentiation of finger activities, such as bending angles at different speeds. This effectively improves stability issues during gesture signal transmission and enables more diverse and precise real-time interaction between human hands and mechanical hands. Regarding vibration sensors, which convert vibration signals from the environment, machinery, or the human body into corresponding electrical signals, challenges arise when designing them based on self-powered TENGs. These challenges include an unstable frequency response to mechanical stimulation, friction layer wear, high structural complexity and manufacturing costs, poor device flexibility, and inadequate safety [41,42,43,44,45]. To address these issues, a fully flexible self-powered vibration sensor was researched and designed (Figure 5b) [35]. As we have seen, given the problems faced by TENG design in the field of self-powered sensing, it is necessary to give priority to the difference and readability of output signals under different conditions during design. Recent research has focused on improving these problems by splitting or coupling the signals and using more sensitive triggers, with significant results.

To better prepare the functional surface of the elastic material, the researchers first chose to introduce laser direct writing technology to fabricate concave laser-induced graphene (LIG) electrodes. Compared to traditional chemical or physical manufacturing methods, laser direct writing onto functional surfaces of elastic materials offers highly convenient, durable, and controllable features. This technology has advantages in rapid, maskless selective cleaning of nanomaterials, adjusting surface properties, or thermal conversion precursors. In this study, instead of using toxic mercury, researchers chose non-toxic Galinstan liquid metal droplets as the oscillator in the device. Its higher surface tension compared to mercury makes it more suitable for contact between the two friction surfaces under various vibrations. As a result, a fully flexible PDMS/Galinstan/LIG-PDMS vibration sensor (working primarily in contact-separation mode) was developed. During vibration, the Galinstan droplets undergo deformation, increasing the contact area with the upper and lower friction layers and generating frictional electrical output. In the field of pressure sensors, the application of TENGs has been reported by many researchers, with the vertical contact-separation working mode widely used due to its suitability for pressure sensor design. These sensors achieve pressure signal-electrical signal output through different voltage states under pressure, thus requiring pressure-driven distance/voltage changes between two friction layers. This has led to various designs, categorized into two types. One type involves structural design to achieve pressure-driven changes in the distance between friction layers, such as arch-shaped substrates [46], spring structures [47], and “mouth”-shaped isolation membranes [48]. Another approach focuses on the design of microstructures on the friction layer surface, aiming to achieve voltage output through pressure-induced vertical displacement of the microstructures on the friction layer. For example, circular/spherical arrays [49,50] and pointed cluster protrusion structures [51] exhibit good pressure responsiveness. Despite the good pressure responsiveness of microstructures, their low output under low-pressure conditions may limit further development. Inspired by fingerprint structures, a variable microstructure dielectric surface was researched and designed (Figure 5c). It works in contact-separation mode. Unlike traditional systems, this asymmetrically paired microelectrode shares a small initial intersection area between the electrodes. When slight pressure causes three-dimensional geometric deformation, the intersection area increases, maximizing its capacitance change and thus maximizing triboelectric generation [36]. Based on the independent layer working mode of TENGs, a TENG driven by a traction belt and a rotating disk has been designed for self-powered pet behavior monitoring and human–pet interaction (Figure 5d) [37]. The entire device consists of a TENG-functionalized pet leash, power management circuitry, and pet wearable electronic devices. It collects mechanical energy from the common movement between pets and owners. Through regulation by the power management circuit, an average output power of 2.38 mW can be obtained at a frequency of 1.5 Hz. During dog walking, the generated energy can continuously drive various pet sensing devices, such as ultrasonic mosquito repellents, motion bracelets, calorie counters, pedometers, etc.

Furthermore, self-powered sensors based on a TENG have been applied in various scenarios. For instance, a wearable human sweat monitoring platform has been developed based on the combination of an electrowetting-on-dielectric (EWOD) device and contact-separation TENG. The EWOD can utilize the high voltage provided by the TENG to achieve the electrowetting effect, allowing sweat droplets to enter the chamber and react with pH indicators for sweat detection [52]. TENGs can be operated using the typical natural/continuous mechanical input source of respiration to design an air detection respirator, powered by the inhalation-driven vertical vibration of the friction layer to obtain electrical output [53]. Moreover, leveraging the high instantaneous power output characteristics of the rotating horizontal sliding mode TENG, a self-powered infrared light wireless communication system has been achieved by introducing optical carriers [54].

### 3.2. Blue Energy

**Figure 6:**
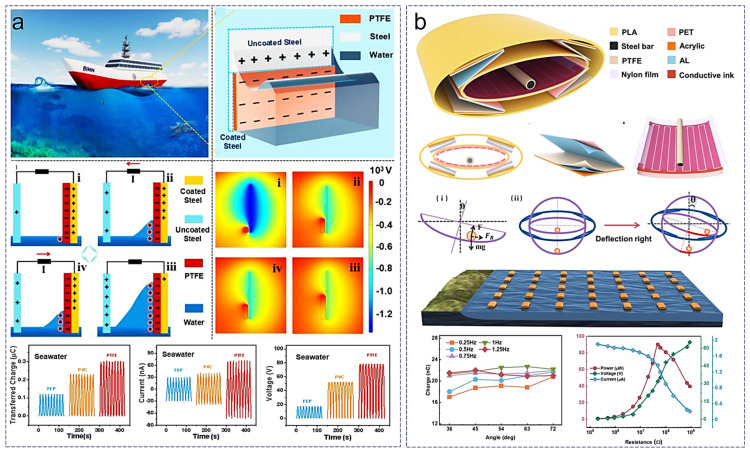
TENGs in blue energy applications. (**a**) Design of a corrosion-resistant dielectric film organic coating to enhance the frictional electricity performance of a water wave TENG [64], (**b**) fully symmetric TENG with an elliptical cylindrical swing structure for wave energy harvesting [65].

Various forms of mechanical energy exist in the natural environment, and the application of TENGs in harvesting natural mechanical energy has been reported by many researchers, such as with raindrop energy [55,56,57] and wind energy [58,59,60,61,62,63]. Among these sources of mechanical energy from the natural environment, the kinetic energy of seawater waves in the ocean is referred to as blue energy. This renewable energy source has been extensively studied due to its widespread distribution, ease of use, high safety, and environmental friendliness. Recently, some innovative designs have emerged in this field (Figure 6).

Traditional ocean wave energy harvesting TENGs are often affected by the side effects of seawater corrosion on the device. Despite the improvement of corrosion resistance, the electrochemical protection method through additional current significantly increases the overall system complexity and application difficulty [66,67,68]. Inspired by commercial ship anticorrosive coatings, a TENG was designed with a dielectric film as an organic coating, covering both coated and uncoated steel shells as two electrodes (Figure 6a) [64]. The dielectric film used in this device exhibited good triboelectric properties and weak ion adsorption effects in seawater due to material optimization, resulting in excellent output performance for the TENG (independent-layer mode). Compared to commercial ship anticorrosive coatings, the dielectric material of this TENG reduced the friction coefficient by approximately half in seawater. This result is beneficial for reducing ship resistance during navigation. Moreover, because the TENG generates a higher voltage for the electrodes during operation, the uncoated steel electrodes exhibit certain anticorrosive properties in seawater, which is crucial for marine energy collection applications. Another challenge in ocean energy collection arises from the instability of sea surface waves. Waves in real environments do not produce fixed size and frequency impacts. Although most previously reported TENGs achieve optimal energy output in turbulent seas, their performance is inadequate in calm seas [69,70,71]. Furthermore, under extreme wave conditions, TENGs may fail to function properly or the output is significantly reduced due to excessive rotation or overturning. Current designs addressing this weakness often sacrifice device flexibility and some output performance [72,73]. To address the motion of ocean waves, a fully symmetrical TENG with an elliptical cylindrical swing structure was designed for wave energy collection (Figure 6b) [65]. This TENG consists of two coaxial elliptical cylindrical shells, with the inner TENG employing a steel bar as an independent mode for the rolling components and the outer TENG comprising four identical contact-separation mode TENGs. The connection rods of the two elliptical cylindrical shells bear bearings. When large waves cause the device to tilt, the contact separation between the inner and outer layers of the TENGs generates output. The elliptical cylindrical design allows the steel rod inside to flexibly roll under small excitations, effectively capturing wave energy on calm sea surfaces. Additionally, the elliptical cylindrical shell has self-stabilizing capabilities and can withstand overturning under severe external forces. Due to its fully symmetrical structure, it can continue to function normally even when overturned by extremely powerful waves, ensuring output performance.

### 3.3. Micro/Nano Power Sources

With the development of the Internet of Things (IoT), countless sensors distributed globally require a power supply, especially in remote areas or harsh outdoor environments where human intervention may be difficult. In such scenarios, sensors need convenient and efficient energy sources to support their autonomous operation. Triboelectric nanogenerators (TENGs) have been studied as micropower sources for self-powered systems due to their simple structure, excellent performance at low frequencies, and portability.

**Figure 7:**
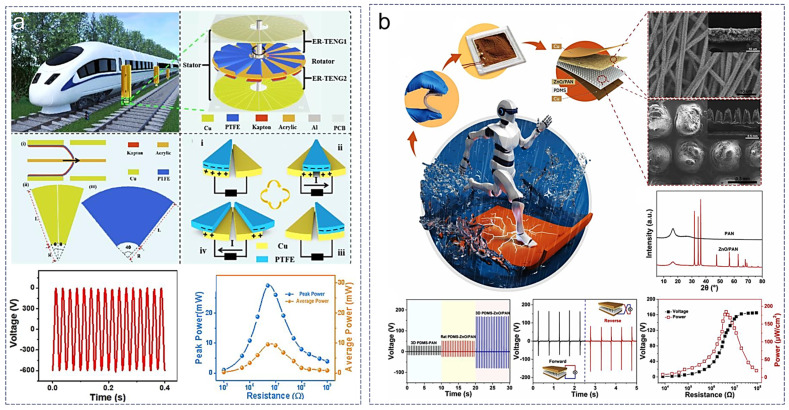
TENGs in micro-nano power supply field. (**a**) Disc-shaped TENG used to harvest wind energy during train travel and power signal devices [74], (**b**) moisture-resistant TENG for outdoor adventure energy supply [75].

One of the high-speed and convenient transportation modes, high-speed rail, often generates wind energy during its operation, which is often overlooked. Moreover, the internal sensing and signaling devices of high-speed trains also require an energy supply. Therefore, a disc-shaped sliding mode TENG designed to collect the wind energy generated during train operation has been developed (Figure 7a) [74]. Considering the severe wear issue of this disc-shaped planar sliding mode TENG, the researchers first selected dielectric layer materials with the lowest friction coefficient through comparison and designed a slightly curved friction layer to achieve elastic contact between the two friction layers. This combination significantly reduces the wear of the TENG during operation, thereby improving the overall energy collection efficiency and service life. Additionally, they adopted a dual TENG combined power generation mode, which further increases the energy conversion efficiency and electrical output performance. The generated electricity can power devices on the train, thus reducing the operating costs of railways. An important application direction of the TENG in micro-nano energy is its use as a power supply for portable wearable electronic devices. This is attributed to the advantages of TENGs over traditional power supply devices such as batteries, including the lightweight, low cost, high safety, ease of integration, environmental friendliness, and high durability [76]. However, most TENGs are affected by various environmental factors, leading to performance degradation, such as due to temperature, humidity, and airborne particles, with humidity exerting a strong inhibitory effect on carrier generation [77]. To address this issue, a waterproof and moisture-proof TENG has been developed through encapsulation to eliminate the influence of humidity on TENG performance (Figure 7b) [75]. This TENG works in contact-separation mode. Device encapsulation typically suppresses the movement distance of the friction layers of contact-separation mode TENGs, inevitably weakening the generation of frictional charge and reducing the output performance. To improve the performance degradation caused by encapsulation, they designed a floral three-dimensional structure on the PDMS surface using laser cutting technology. These three-dimensional protrusions help expand the contact area during contact, facilitate rapid electron transfer, and enhance the overall output performance. This waterproof and moisture-proof TENG enables the collection and conversion of motion energy during outdoor activities in humid environments, while the patterned three-dimensional structure provides sufficient electrical output to power portable electronic devices.

### 3.4. HV Power Sources

In addition to the portability and simplicity mentioned above, a TENG also possesses an important inherent output characteristic, high output voltage and low output current. A TENG can generate kilovolt-level high voltage without complex conversion devices. Moreover, due to the limitation of transferred charge within each cycle, despite its low current output, this feature can also reduce safety hazards for both instruments and operators [78,79,80].

**Figure 8:**
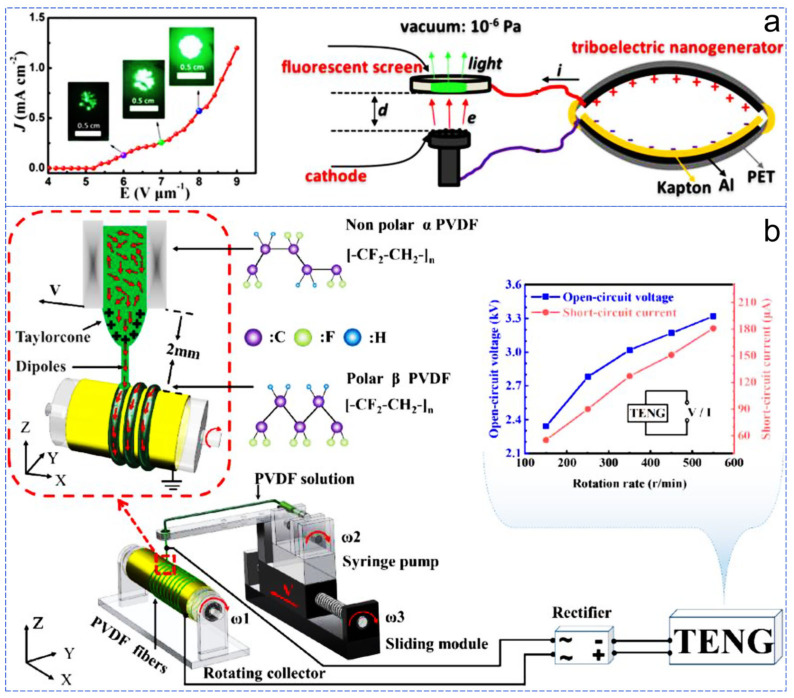
TENG in high voltage power supply field. (**a**) TENG-driven field emission device [81], (**b**) TENG-driven electrospinning apparatus [82].

Field electron emission is the process in which electrons are emitted from the surface of a material under the influence of an applied electric field through quantum mechanical tunneling. Field emission devices are typical high-voltage power-driven devices that require continuous high-voltage power to achieve high current density and stable electron emission. However, conventional high-voltage power supplies are usually bulky and heavy, severely limiting the portability of devices and their application in modern miniaturized electronic devices. A simple structured contact-separation mode TENG capable of generating high voltage can be used to drive field emission devices, offering the prospect of preparing portable field emission devices (Figure 8a) [81]. Electrospinning is a technique for precisely depositing fibers and various complex two-dimensional and three-dimensional fiber structures, but it typically requires kilovolt-level high-voltage power for driving. Currently, although commercial high-voltage power supplies (AC/DC) can meet the voltage requirements, they come with drawbacks in terms of cost and energy consumption, increase the overall complexity of the equipment, and pose potential threats to the life safety of operators. Therefore, replacing traditional high-voltage power supplies with TENG power is an effective approach (Figure 8b). By combining TENGs with near-field electrospinning technology, the precursor solution of PVDF on the needle tip is continuously deposited on the rotating drum electrode over a short distance (2 mm), resulting in ordered PVDF fibers without additional polarization and stretching [82].

## 4. Improvement Strategies for Performances

Despite the broad application prospects, the TENG’s output performance still needs further improvement due to its inherent megaohm-level impedance. Based on TENG’s working principle, methods for enhancing TENG performance can be roughly categorized into three types: designing energy management circuits, improving the overall or microstructure of the device, and modifying the friction layer materials.

### 4.1. Energy Management

Due to the often unstable and irregular mechanical energy in the TENG’s application scenarios, the output amplitude and frequency tend to be random, posing challenges for energy utilization and storage. Additionally, the TENG’s inherent high impedance may mismatch with electronic devices or energy storage units, significantly impacting the efficiency of energy utilization from the TENG’s output [83]. Traditional energy management methods are inadequate to handle this irregular phenomenon; hence, the development of efficient energy management circuits is crucial.

**Figure 9:**
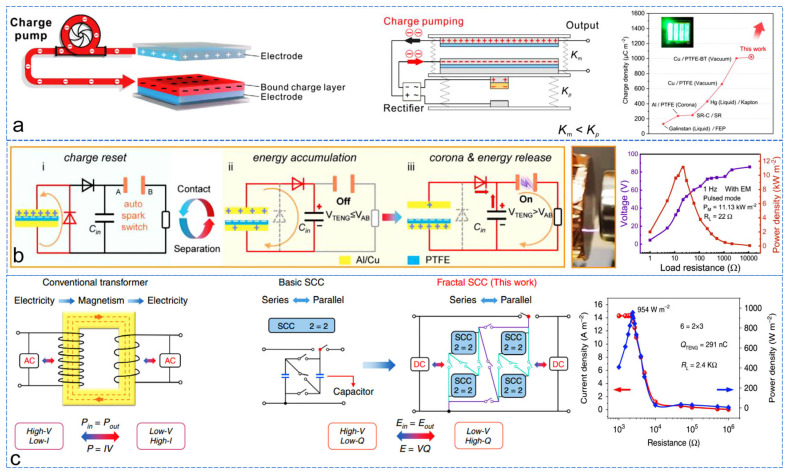
Enhancing TENG output performance through energy management. (**a**) Increasing TENG surface charge density using a charge pump [84], (**b**) energy management circuit based on spark switches with full-range voltage availability [85], (**c**) energy management circuit based on switch-capacitor transformers with fractal structure [86].

Improving the output performance of a TENG through energy management can generally be achieved through two approaches. One approach is to increase the charge quantity. The surface charge density of the TENG directly affects its power density output. Therefore, injecting electrons directly into a TENG can enhance the energy by increasing its charge quantity. A structurally simple and versatile electron injection charge pump has been designed to achieve electron injection and enhance the output performance of a TENG (Figure 9a) [84]. Due to the use of a contact-separation working mode, something similar to the TENG’s charge pump can be integrated with a friction motor to form a double-layer charge-injection TENG, further simplifying the structure. This integrated device can achieve an effective surface charge density of 1020 μC/m^2^ during contact-separation mode operation, which is four times the air breakdown density, demonstrating significant performance improvement. Another approach is to design appropriate energy management circuits, which can improve output performance through voltage transformation [87], rectification filtering [88,89], energy storage, and instantaneous closing/opening switches [90,91]. Circuit switches combined with transformer design are important for accumulating low-frequency signals and releasing them in high-frequency form. However, most switches cannot operate reliably under voltages exceeding 1000 V and may experience leakage current under environmental interference. Traditional transformers cannot match the TENG’s variable output amplitude and frequency. Nevertheless, using spark discharge effects can achieve a design of automatic spark switches with very low leakage current and a wide range of applicable voltages (Figure 9b) [85]. This TENG works in contact-separation mode. The spark switch opening voltage is adjustable and can rapidly convert low-frequency energy collected by the TENG into high-frequency energy. With an air gap of only 2.4 mm, the TENG can provide a high voltage of 7.5 kV to open the switch, far exceeding the performance of traditional energy management work. They also provide a design scheme for induction transformers that can match random low-frequency electrostatic energy. With these two combined, their energy management strategy achieves a high-power density of 11.13 kW/m^2^ (TENG area of 0.01 m^2^) and an excellent stable output capability, with an average power output of 1 mW/Hz. Working at a frequency of 1 Hz can continuously drive a 4 × 4 sensor array. Furthermore, switch-capacitor converters are favored by some researchers due to their integration, lack of magnets, and lightweight compared to traditional transformers. For the high output impedance and switch losses of switch-capacitor transformers, a fractal structure capacitor-type energy management scheme has been studied for output performance (Figure 9c) [86]. The fractal design is achieved through the self-replacement of multiple basic switch-capacitor transformation units, with each fractal conversion unit composed of a capacitor connected by diodes. This design effectively reduces the total output voltage drop of the system, improves the operational efficiency, and achieves a power density of 954 W/m^2^ and an energy transfer efficiency of over 94% in pulse mode.

As an easy-to-operate and low-cost strategy to effectively improve the output performance of the TENG, circuit design has the disadvantage of increasing the overall structural complexity and space volume ratio, which undoubtedly hinders the expansion of its application scenarios. Therefore, an efficient circuit design strategy requires a combination of performance improvement and overall structural simplification.

### 4.2. Structural Improvement

Improving the structure of the TENG can enhance not only its output performance but also the overall mechanical properties of the device, such as robustness and wear resistance. Generally, structural improvements can be classified into two approaches: improving the overall working structure of the device and designing the microstructure of the friction layers’ surface.

**Figure 10:**
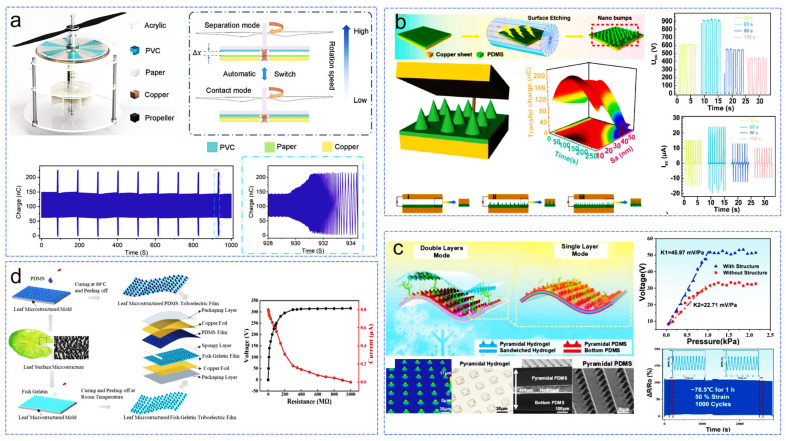
Enhancing TENG output and wear resistance through structural improvements. (**a**) Aerodynamic structural design to enhance TENG wear resistance [92], (**b**) surface etching to create various nanoscale protrusion structures on the friction layer [93], (**c**) dual-network organic ion hydrogel with surface micro-pyramid structures [94], (**d**) gelatin-based TENG inspired by leaf surface microstructures [95].

Designing structural improvements for TENGs can reduce operational wear while maintaining high energy output and stability (Figure 10a) [92]. In this study, based on aerodynamics, the combination of propellers and rotors allows TENGs to transition from contact mode to separation mode at a certain rotational speed. The authors chose to optimize the stiffness coefficient of the spring to reduce the rotational speed required for separation, enabling the designed TENG to freely switch between working modes above and below the rotational speed threshold, ensuring the replenishment of surface charges. Even after 138.7 cycles, it still maintained over 95% of its output, demonstrating excellent stability. Other researchers aim to enhance output performance by designing microstructures on the surface of the friction layers to increase the interlayer contact area. Among them, a series of nano-protrusions (10–30 nm) prepared on the friction layer surface using surface etching technology showed promising performance improvement (Figure 10b) [93]. A high voltage and current output of 910 V and 24 μA were achieved after 60 s of etching, demonstrating significant performance enhancement. Tao et al. [94] introduced a combination of surface micro-pyramid structured double network organic ionic hydrogel and PDMS friction layers spin-coated/deposited on micro-pyramid etched silicon wafers in TENGs (contact-separation mode), achieving high transparency (85%), sensitivity (45.97 mV/Pa), and response time (20 ms), along with excellent durability and stability (Figure 10c). Inspired by leaf surface structures, Shi et al. designed a micro-structured TENG (contact-separation mode) based on fish gelatin (Figure 10d) [95]. Among the four designed structures, the micro-pyramid structure mimicking lotus leaves achieved the highest performance improvement, with the voltage and current reaching 320 V and 0.8 μA, respectively, representing 5.8 times and 32 times improvement over the non-micro-structured counterpart.

The rotating disk (horizontal sliding mode) TENGs are usually designed to operate at high rotational speeds to collect more energy, but the inevitable material wear resulting from high-speed operation leads to a decline in electrical output performance [96,97]. Although TENGs can operate in a non-contact mode after being energized in contact mode, performance degradation due to surface charge dissipation needs to be considered [98]. Although there are many types of surface microstructures, most require complex fabrication processes and higher costs [99,100]. In the structural design strategy, the whole structure design has similar disadvantages to the energy management circuit design, which increases the complexity of the whole structure. The increased complexity and space volume ratio will reduce the application scope of the TENG to a certain extent. Although the surface structure design reduces the impact on the overall structure, the operation process is often more complex and requires higher costs. Therefore, although the structural design strategy can be optimized in more aspects, such as the output performance and service life, the design needs to simplify the overall structure, control the manufacturing cost, and reduce the difficulty of operation, such as selecting the structural design method that can be mass-manufactured (laser processing, electrostatic spinning, etc.).

### 4.3. Material Modification

Among the various improvement methods, both energy management and structural design have certain limitations to performance enhancement when their utilization alone. Energy management can only optimize output performance, and its effect is relatively singular. It cannot fundamentally solve TENG’s poor performance issues from the perspective of material properties. Moreover, whether it is the addition of electron injection devices or energy management circuits, they all increase the overall complexity of the structure. On the other hand, in the structural design, while enhancing the service performance of the device, it often reduces the triggering frequency. In contrast, the design of interface microstructures can directly act on the contact interface, with flexible structures and diverse sizes, exerting minimal impact on the energy density of TENGs. However, the direction of performance optimization in structural design also has limitations. In comparison, the selection and modification of friction layer materials can optimize the contact interface based on the material properties, thereby enhancing various performance aspects for TENG operation. This approach has high applicability and wide-ranging applications.

**Figure 11:**
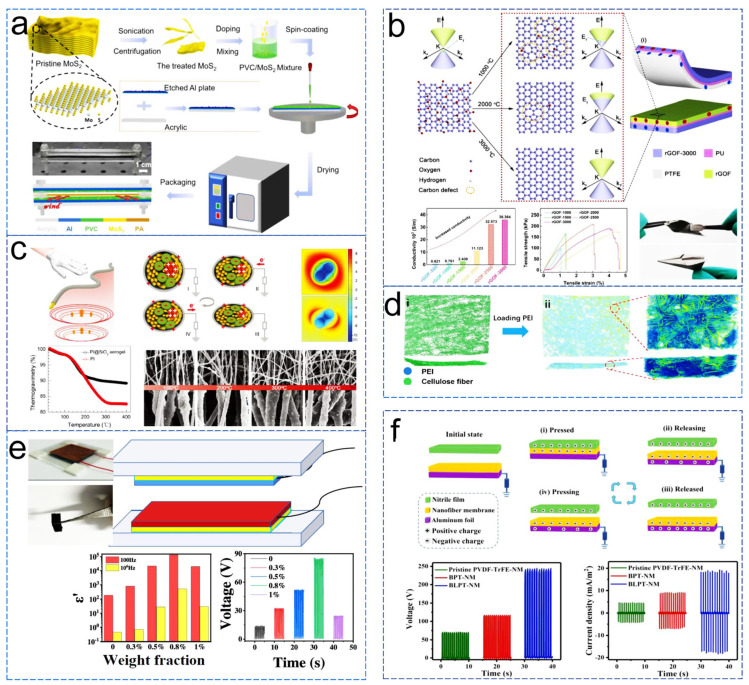
Enhancing TENG performance through material modification. (**a**) PVC/MoS2 composite film with high wear resistance and output performance [101], (**b**) defect-mediated strategy for adjusting the work function [102], (**c**) high-temperature resistant aerogel-coated twisted friction nanofibers [103], (**d**) method of enhancing cellulose paper frictional properties via loading PEI [104], (**e**) performance enhancement strategy via regulating MWCNT composite content to increase film dielectric constant [105], (**f**) performance enhancement strategy via composite high-κ doped barium titanate to increase dielectric constant [106].

High-speed operation of TENGs under high-output modes presents challenges in their lifespan, as inevitable surface wear leads to decreased performance. A friction layer material with excellent wear resistance was prepared by compounding PVC with molybdenum disulfide (Figure 11a). This TENG works in contact-separation mode. Molybdenum disulfide, acting as a solid lubricant, simultaneously enhances the surface charge density of the film, achieving dual improvements in wear resistance and output performance, ultimately obtaining excellent voltage and current outputs of 398V and 40uA, respectively [101]. Adjusting the work function is an effective method to enhance the gain and reduce the electron capabilities of materials. Therefore, a defect-mediated strategy for adjusting the work function of graphene materials was studied and applied to improve the output performance of TENGs (Figure 11b) [102]. By obtaining different oxygen and carbon defect contents through annealing at temperatures ranging from 500 °C to 3000 °C, the work function of graphene friction materials could be continuously and widely adjusted from 4.68 eV to 4.49 eV. Among them, reduced graphene oxide annealed at 2000 °C exhibited fewer oxygen defects and more carbon defects, resulting in the lowest work function, which was conducive to minimizing electron losses in the positive friction materials. This TENG works in contact-separation mode, the output voltage and current were increased to 190 V and 14 μA, respectively, and the power density reached 5.04 W/m^2^. One of the factors limiting TENG’s performance is the environmental conditions. TENGs often fail to achieve the desired output at high temperatures. Therefore, a nano-coated high-temperature-resistant frictional electric nanofiber was studied and designed (Figure 11c). The authors not only composed the TENG (contact-separation mode) with twisted fiber structures but also significantly increased the applicable temperature limit by introducing silica aerogel [103]. Cellulose paper exhibits excellent positive electricity, making it suitable as a friction layer material for TENGs. However, its relatively low output performance still requires further enhancement. By loading branched polyethyleneimine (PEI), the frictional electrical performance of cellulose paper can be effectively improved (Figure 11d) [104]. By loading 7.5 mg/cm^2^ of PEI to form a network structure and 22.5 mg/cm^2^ of PEI to form a hydrogel structure, the output performance of the contact-separation mode TENG (charge transfer, open-circuit voltage, and current) was increased by approximately 4 times and 6 times, respectively. According to the frictional power generation theory model proposed by academician Zhonglin Wang, the dielectric constant of the friction layer material has a significant impact on the output performance of TENGs. Therefore, the dielectric properties of frictional materials are extensively studied to enhance output performance. Han et al. [105] designed a TENG (contact-separation mode) based on a multi-walled carbon nanotube (MWCNT)/chitosan (CS) composite film (Figure 11e). Adding MWCNT as a conductive filler effectively increased the dielectric constant of the film before reaching the percolation threshold. When the highest dielectric performance was achieved, the voltage, current, and charge transfer reached 85.8 V, 8.7 μA, and 29 nC, respectively, which were 82, 31, and 5 times higher than the original values. The maximum output power density reached 180 mW/m^2^ (50 MΩ load). Additionally, Xi et al. [106] synthesized BaTiO_3_: La nanoparticles with a higher dielectric constant than pure barium titanate using a hydrothermal method and optimized the content of BaTiO_3_: La and the doping concentration of La. They prepared BaTiO_3_: La/PVDF-TrFE nanofiber composite membranes through electrospinning and assembled single-electrode mode TENGs. The device exhibited an output voltage of 245V, a friction charge density of 87.3 μC/m^2^, and a maximum instantaneous power density of 2.52 W/m^2^, demonstrating significant performance improvements (Figure 11f).

Based on the above research content and the current development trend of TENG performance optimization, material selection first tends to improve the triboelectric polarity difference to improve the surface charge generation efficiency. When modifying the material, it is necessary to give priority to improving the surface charge density, which is one of the important factors in the output performance of the TENG. Therefore, the selection of high dielectric performance fillers, such as ferroelectric fillers, under the premise of controlling dielectric loss is a very effective output enhancement strategy. Dielectric properties not only affect the surface charge density of the friction layer, but also affect the air breakdown strength. Materials with high air breakdown strength have an excellent charge retention rate to inhibit the escape of charge. In addition, special modifications can be made to the material according to the application requirements in different environments, such as the mixture of high temperature/low temperature resistant materials.

## 5. Conclusions and Challenges

### 5.1. Summary

The electrical output principles of friction-based nanogenerators are continuously evolving and gradually being established and extended. There are four proposed operating modes, and the materials have also evolved from solid to liquid. The diversity of operating modes and the availability of friction electrode materials facilitate the design and research of friction-based nanogenerators and bring potential possibilities for practical application scenarios.

Among the four main application directions, self-powered sensors contribute to the increased proliferation of future microelectronic networks, while battery-free sensors will expand their practical application range. Blue energy, as a collector of marine energy, has significant potential for sustainable development, especially after the gradual exploration of the frictional electricity principle in liquid materials. Micro-nano energy focuses on using TENGs primarily as energy harvesting devices, enabling further collection and utilization of natural environmental energy and weak mechanical energy. Research on high-voltage power supplies benefits many high-voltage demand devices, which is also an effective utilization of the TENG’s high-voltage, low-current output.

The output performance of the TENG still falls within the realm of relatively weak energy devices. Among the three main directions for optimizing output performance, energy management tends to focus on circuit design. Although the operation is relatively convenient, it inevitably increases the complexity and space requirements of the device. Structural design improvements, on the other hand, involve macroscopic changes that can be optimized according to specific application requirements or service life demands, but they still add complexity to the device. Surface microstructure design tends to have a smaller impact on the overall volume of the device and offers greater operational space, but actual fabrication requires the corresponding equipment, leading to higher costs. Material modification aims to enhance certain properties of the friction layer based on the material response’s attributes to improve output, which is beneficial for developing microelectronic devices. Furthermore, there is ample room for improvement in this area, and with advancements in material science, there will be increasing possibilities. However, the process of material modification is complex. It is necessary to comprehensively consider the practical feasibility of various methods to achieve better performance improvement results in the actual performance optimization.

### 5.2. Challenges and Prospects

Although the TENG underwent rapid development since its proposal in 2012, with significant contributions from numerous researchers leading to advancements in performance and device design, some significant issues cannot be overlooked. Firstly, the TENG faces challenges related to device stability and lifespan, during its practical application and promotion. The majority of the TENG’s output performance relies on direct material contact, which can lead to issues with material lifespan. Additionally, there is a risk of variations in the effective contact area under the same force and frequency during actual contact processes, resulting in minor fluctuations in performance. Secondly, further improvement is needed in the TENG’s output performance. The high-voltage and low-current output characteristics combined with the inherently high impedance (megaohms) limit its practical application scenarios due to low output power. As research progresses, the TENG holds promise for breakthroughs in existing performance enhancement directions as well as new avenues for performance improvement. These breakthroughs include not only enhancements in performance but also improvements in stability and sustainability in practical use. Ultimately, the TENG has the potential to achieve batch production and widespread use corresponding to real-world applications. The TENGs with high sensitivity and no need for an external power supply, should be further explored to promote their application possibilities under extreme conditions, such as high temperature, high pressure, high humidity, and low temperature. In addition, as one of the nanogenerators driven by mechanical energy, if the TENG wants to achieve deep functional expansion in future development, it should consider whether it can convert from electrical energy to mechanical energy in special scenarios, such as a complete vacuum environment, which will promote the field to expand in a new direction.
